# Dinucleotide repeat polymorphism in Fms-like tyrosine kinase-1 (Flt-1) gene is not associated with preeclampsia

**DOI:** 10.1186/1471-2350-9-68

**Published:** 2008-07-17

**Authors:** Shin-Young Kim, Ji-Hyae Lim, Jae-Hyug Yang, Moon-Young Kim, Jung-Yeol Han, Hyun-Kyong Ahn, Jun-Seek Choi, So-Yeon Park, Mi-Jin Kim, Hyun-Mee Ryu

**Affiliations:** 1 Laboratory of Medical Genetics, Cheil General Hospital and Women's Healthcare Center, Kwandong University College of Medicine, Seoul, Korea; 2 Department of Obstetrics and Gynecology, Cheil General Hospital and Women's Healthcare Center, Kwandong University College of Medicine, Seoul, Korea

## Abstract

**Background:**

Preeclampsia is a major cause of maternal and perinatal mortality and morbidity. The etiology of preeclampsia remains unclear. Recently, it was shown that misregulation of fms-like tyrosine kinase-1 (Flt-1) in the peripheral blood mononuclear cells of pregnant women results in over-expression of the soluble splice variant of Flt-1, sFlt-1, producing an additional (extra-placental) source of sFlt-1 that can contribute to the etiology of preeclampsia. The aim of this study was to investigate the relationship between preeclampsia and a dinucleotide (threonine-glycine; TG)_n _repeat polymorphism in the 3' non-coding region of the Flt-1 gene.

**Methods:**

The number of the d(TG)_n _repeats was analyzed in 170 patients with preeclampsia and in 202 normotensive pregnancies. The region containing the dinucleotide repeat polymorphism of the Flt-1 gene was amplified by polymerase chain reaction (PCR) from the DNA samples and was analyzed by direct PCR sequencing.

**Results:**

We found 10 alleles of the dinucleotide repeat polymorphism and designated these as allele*12 (A1) through allele*23 (A12) according to the number of the TG repeats, from 12 to 23. The frequency of the 14-repeat allele (A3) was most abundant (63.82% in preeclampsia and 69.06% in controls), followed by the 21-repeat allele (A10; 28.53% in preeclampsia and 23.76% in controls). There was no significant difference in the allele frequency between patients with preeclampsia and normal controls. The most common genotype in preeclamptic and normotensive pregnancies was heterozygous (TG)_14_/(TG)_21 _(41.76%) and homozygous (TG)_14_/(TG)_14 _(45.05%), respectively. However, the genotype frequencies were not significantly different between preeclamptic patients and controls.

**Conclusion:**

This is the first study to characterize the dinucleotide repeat polymorphism of the Flt-1 gene in patients with preeclampsia. We found no differences in the allele or genotype frequencies between patients with preeclampsia and normal pregnancies. Although limited by a relatively small sample size, our study suggests that the d(TG)_n _repeat polymorphism of the Flt-1 gene is not associated with the development of preeclampsia in Korean pregnant women.

## Background

Preeclampsia, a pregnancy-specific syndrome affecting about 5% to 10% of all pregnancies, is characterized by hypertension and proteinuria after 20 weeks of gestation. It is a major cause of maternal and perinatal morbidity and mortality and occurs only in the presence of a placenta and remits dramatically after the placenta has been delivered. The underlying pathogenetic mechanisms of this maternal syndrome are much debated with the current hypotheses including inflammatory disease, vascular-mediated factors, placental ischemia, genetic predisposition and immune maladaptation [1-3]. Preeclampsia is also associated with defective uteroplacental vascularization [4] and impairment of angiogenesis and vascular transformation of the uteroplacental unit, which are crucial for normal fetal development [5]. However, the molecular pathways responsible for normal angiogenesis and vascular remodeling in the fetomaternal unit are still poorly understood.

Vascular endothelial growth factor (VEGF) is a major angiogenic factor and plays an important role in all aspects of vascular development, including endothelial cell proliferation, migration, survival and regulation of vascular permeability [6,7]. VEGF and its two receptors, fms-like tyrosine kinase 1 (Flt-1) (VEGFR-1) and KDR/Flk-1 (VEGFR-2), have been shown to be part of an essential regulatory system for blood vessel formation. One of the two receptors, Flt-1 appears to have dual functions, with negative and positive activity in vascular endothelial cells. In early embryogenesis, Flt-1 functions as a negative regulator, most likely through its strong VEGF trapping activity [8]. Flt-1 (-/-) mice are embryonic lethal due to the disorganization of blood vessels and overgrowth of endothelial-like cells within the lumens of blood vessels [9]. In adult stages, however, Flt-1-specific ligand can induce a mild angiogenesis. Thus, this dual function may be tightly regulated and important for fine tuning the formation and maintenance of the blood vessel structure in placental vasculature.

During placental development, the expression of the Flt-1 gene is not only detected in vascular endothelial cells, but also in the developing trophoblasts [10]. The Flt-1 gene is located in the chromosome region 13q12 and consists of 30 exons and 29 introns [11,12]. The gene encodes a high affinity receptor for VEGF, which has sixth immunoglobulin (Ig)-like domains in the extracellular region based on the distribution of cysteine residues, a transmembrane domain and an intracellular region containing a tyrosine kinase domain divided by a long kinase-insert domain [11,13]. There is a dinucleotide repeat in the 3' non-coding region of the Flt-1 gene [11] that has been shown to be polymorphic [14,15]. This region of the gene codes for the intracellular part of the protein that is likely associated with signal transduction. Dinucleotide repeat regions are often used as disease markers and their functional significance is being increasingly realized [16,17]. Considering the important roles of Flt-1 in pregnancy, functional polymorphisms in the Flt-1 gene may be potentially important as genetic markers for susceptibility to preeclampsia. Based on genetic predisposition, this relationship may be strengthened by showing an association between polymorphisms of Flt-1 gene and an increased risk of developing preeclampsia. In view of the possible role of the Flt-1 gene in the etiology of preeclampsia, we investigated whether the dinucleotide (threonine-glycine; TG)_n _repeat polymorphism in the 3' non-coding region of the Flt-1 gene is associated with preeclampsia in Korean pregnant women.

## Methods

### Subjects

All subjects were recruited from the Obstetrics and Gynecology Department at Cheil General Hospital between October 2001 and June 2004. The study population included 170 patients with preeclampsia (13 mild and 157 severe) and 202 sampling week-matched normal pregnancies of Korean origin. Preeclampsia was defined as hypertension (systolic blood pressure ≥ 140 mmHg and/or diastolic blood pressure ≥ 90 mmHg after 20 weeks' gestation) and proteinuria (≥ 300 mg in a 24 h urine collection or ≥ 1+ on dipstick testing) according to the criteria of the National High Blood Pressure Education Program Working Group Report on High Blood Pressure in Pregnancy [18]. Severe preeclampsia was diagnosed on the basis of diastolic blood pressure ≥ 110 mmHg or significant proteinuria (dipstick measurement of ≥ 2+) or the presence of headache, visual disturbances, upper abdominal pain, oliguria, convulsion, elevated serum creatinine, thrombocytopenia, marked liver enzyme elevation or pulmonary edema [18]. Only one woman was a HELLP syndrome in preeclamptic patients. Exclusion criteria included major congenital anomalies, fetal chromosomal abnormalities, chronic hypertension, diabetes or renal disease. Controls were selected randomly from contemporaneous women who were normotensive and who were without proteinuria throughout pregnancy, and who delivered a healthy neonate at term without significant medical or obstetric complications (including intrauterine growth retardation; IUGR). IUGR was defined as a birth weight less than the 10th percentile for gestational age. Written informed consent was obtained from all subjects before blood sampling, which was approved by the Ethics Committee of Cheil General Hospital and Women's Health Care Center.

### DNA Sequencing

Peripheral blood was collected in EDTA vacutainer tube (Becton Dickinson, USA) for genomic DNA isolation. Genomic DNA was extracted from whole blood using a QIAamp DNA Blood Mini Kit (Qiagen GmbH, Hilden Germany) according to the manufacturer's recommendations. A genomic fragment containing the d(TG)_n _repeat polymorphism of the Flt-1 gene was amplified by polymerase chain reaction (PCR) using the fluorescent 6FAM-labelled forward primer 5'-6FAM-TTTGGCCGACAGTGGTGTA-3' and the unlabelled reverse primer 5'-CTTTAAAATTCCAGTTTCCTT-3', according to Parry [19]. PCR reactions were performed in a final volume of 20 ul containing 10 ng of genomic DNA, 2 ul of 10× PCR buffer (Applied Biosystems, Foster City, CA, USA), 1.25 mM MgCl_2 _(Applied Biosystems), 1 mM of each dNTP (Applied Biosystems), 10 pmol of each specific primer and 1 U of Taq DNA Polymerase (Applied Biosystems). The PCR conditions consisted of an initial denaturation at 95°C for 5 min, followed by 33 cycles of denaturation at 95°C for 40 s, annealing at 55°C for 40 s, extension at 72°C for 40 s, and a final extension at 60°C for 30 min on ABI PRISM 2700 thermal cycler (Applied Biosystems). An aliquot of the amplified PCR product was electrophoresed on a 3% neusive gel to verify product quality and quantity.

PCR products were purified with AccuPrep^® ^PCR purification kit (BIONEER, Korea) and the purified PCR products were sequenced with an ABI PRISM BigDye Terminator Cycle Sequencing Kit (Applied Biosystems). The number of TG repeats within each allele was analyzed by direct sequencing using an ABI PRISM 3100 Genetic Analyzer (Applied Biosystems). The electropherogram traces were interpreted by Genescan software version 3.7 (Applied Biosystems) and corresponding genotypes assigned using Genotyper software version 3.7 (Applied Biosystems). All samples were analyzed twice to confirm the integrity of the results.

### Statistical Analysis

Data were expressed as the mean ± SD, median (range) or number (%). The Student's *t*-test was used for comparison of continuous variables between normal controls and preeclamptic patients. Categorical variables, such as the comparison in the allele and genotype frequencies between the two groups, were analyzed by a 2 × 2 Chi-Squared test or Fisher's exact test. Odds ratios (OR) and 95% confidence intervals (CI) were calculated to assess the disease risk conferred by genotypes. Hardy-Weinberg equilibrium was tested in patients 0.05 was considered statistically significant. and controls, separately, by means of the Chi-Squared analysis . A *p *value < 0.05 was considered statistically significant. The statistical analysis was performed with the Statistical Package for Social Sciences version 10.0 (SPSS Inc., Chicago, USA).

## Results

Polymorphism analysis of the Flt-1 gene was performed for 170 preeclamptic patients and 202 normotensive pregnancies. The clinical characteristics of the study population are shown in Table 1. Maternal age, nulliparity, blood pressure, maternal weight at delivery, gestational age at delivery and birth weight were found to be significantly different between the two groups (*p *< 0.05). In addition, 58 (34.12%) of the 170 preeclamptic patients delivered a fetus with IUGR, defined as birth weight below the 10th percentile for gestational age.

**Table 1:** Clinical characteristics of normal controls and preeclamptic patients

Characteristics	Controls (n = 202)	Patients (n = 170)	*p *value*
Maternal age (y)	32.8 ± 3.7	30.9 ± 3.8	< 0.001
Nulliparity (%)	94 (46.5)	139 (81.8)	< 0.001^†^
Systolic BP (mmHg)	121.8 ± 10.2	159.2 ± 16.6	< 0.001
Diastolic BP (mmHg)	75.3 ± 8.7	100.5 ± 11.8	< 0.001
Maternal weight at delivery (kg)	68.0 ± 7.8	74.2 ± 10.3	< 0.001
GA at delivery (wk)	39.1 ± 1.3	36.5 ± 3.3	< 0.001
Birth weight (g)	3356.9 ± 436.1	2528.0 ± 776.4	< 0.001
Proteinuria (dipstick)	-	2.6 ± 1.0	-
IUGR (number)	-	58	-

BP = blood pressure; GA = gestational age; IUGR = intrauterine growth retardation

A *p *value was calculated using the *Student's *t*-test or ^†^Chi-Squared test.

Significant value was taken at the level of *p *< 0.05.

Ten alleles observed in this study groups were designated as allele*12 (A1) through allele*23 (A12), according to the number of TG repeats, which ranged in size from 102 bp (12 TG repeats with a 78 bp segment of amplified flanging sequences) to 124 bp (23 TG repeats) (Table 2). The frequency of the 14-repeat allele (A3) was most abundant (63.82% in preeclampsia and 69.06% in controls), followed by the 21-repeat allele (A10; 28.53% in preeclampsia and 23.76% in controls) (Table 2). The allele frequency was very similar to that previously reported for an American population [14]. Interestingly, the 12-, 15-, 19-, and 20-repeat alleles had not previously been reported. However, the allele frequencies of the Flt-1 (TG)_n _polymorphism in patients with preeclampsia did not differ from those in normal controls. Furthermore, the genotype frequencies in preeclampsia and controls did not significantly deviate from Hardy-Weinberg equilibrium (data not shown). The genotypes were classified into five groups; (TG)_14_/(TG)_14_, (TG)_14_/(TG)_21_, (TG)_21_/(TG)_21_, Other, Combination (Table 3). The most common genotype in normal controls (45.05%) was homozygous (TG)_14_/(TG)_14_, whereas those in preeclamptic patients (41.76%) was heterozygous (TG)_14_/(TG)_21 _(Table 3). We compared each reference group [(TG)_14_/(TG)_14_, (TG)_14_/(TG)_21_, or (TG)_21_/(TG)_21 _genotype] with Other (Table 3). Additionally, we also compared (TG)_14_/(TG)_14 _genotype with the combined genotype [(TG)_14_/(TG)_21_+(TG)_21_/(TG)_21_+Other] (Table 3). However, the genotype frequencies of the Flt-1 (TG)_n _polymorphism in preeclamptic patients did not differ from those in normotensive pregnancies.

**Table 2:** Allele frequencies of the Flt-1 (TG)_n _polymorphism in normal controls and preeclamptic patients

Allele	TG repeat	Controls	Patients
		
	(PCR product length, bp)	2n (%)	2n (%)
A1	12 (102)	2 (0.50)	0 (0.00)
A2	13 (104)	4 (0.99)	4 (1.18)
A3	14 (106)	279 (69.06)	217 (63.82)
A4	15 (108)	3 (0.74)	2 (0.59)
A5	16 (110)	1 (0.25)	0 (0.00)
A6	17 (112)	0 (0.00)	0 (0.00)
A7	18 (114)	0 (0.00)	0 (0.00)
A8	19 (116)	5 (1.24)	3 (0.88)
A9	20 (118)	2 (0.50)	6 (1.76)
A10	21 (120)	96 (23.76)	97 (28.53)
A11	22 (122)	8 (1.98)	7 (2.06)
A12	23 (124)	4 (0.99)	4 (1.18)

**Table 3:** Genotype frequency of the Flt-1 (TG)_n _polymorphism in normal controls and preeclamptic patients

Genotype	Controls	Patients	OR (95% CI)	*p *value
			
	n (%)	n (%)		
(TG)_14_/(TG)_14_	91 (45.05)	65 (38.24)	1.11 (0.59–2.09)	0.746^a^
(TG)_14_/(TG)_21_	76 (37.62)	71 (41.76)	0.85 (0.45–1.60)	0.613^b^
(TG)_21_/(TG)_21_	6 (2.97)	11 (6.47)	0.43 (0.14–1.35)	0.143^c^
Other	29 (14.36)	23 (13.53)	-	-
Combination	111 (54.95)	105 (61.77)	1.32 (0.87–2.01)	0.185^d^

OR = odds ratios; CI = confidence interval.

Other, any genotypes other than the (TG)_14_/(TG)_14_, (TG)_14_/(TG)_21_, or (TG)_21_/(TG)_21 _genotype.

Combination, combined genotype [(TG)_14_/(TG)_21_+(TG)_21_/(TG)_21_+Other].

A *p *value was calculated using the Chi-Squared test or Fisher's exact test.

Significant value was taken at the level of *p *< 0.05.

^a^(TG)_14_/(TG)_14 _versus Other.

^b^(TG)_14_/(TG)_21 _versus Other.

^c^(TG)_21_/(TG)_21 _versus Other.

^d^(TG)_14_/(TG)_14 _versus Combination.

## Discussion

Microsatellites are powerful tools for performing linkage and association studies for diseases but they may also be directly involved in the modification of gene expression levels by silencing/enhancing transcription and modulating splicing events [20,21]. Since the microsatellite is located within intronic sequence with no obvious functional relevance, as shown by Turecki [22], it is possible that specific alleles of this repeat may be in linkage disequilibrium with a nearby polymorphism that affects disease susceptibility. Moreover, variability in simple intronic repeats is probably involved in the etiology and pathogenesis of multifactorial diseases [23].

However, although genetic factors have been extensively investigated during the past decade [24], the dissection of the genetic basis for preeclampsia has been challenged by the wide clinical heterogeneity of this disorder and the lack of a full understanding of its underlying cause. The genes expressed in the placental vasculature throughout pregnancy are attractive candidate genes and nearly all accessible genes have been extensively analyzed. The Flt-1, a major receptor for VEGF, is produced as a 1338 amino acid residue precursor with a predicted 22 amino acid signal peptide. The extracellular domain of Flt-1 is composed of 736 amino acids; its transmembrane spanning domain is 22 amino acids and its intracellular domain is 558 amino acids. Spongiotrophoblast cells, endothelial cells and their progenitors are the major placental source of Flt-1 [25,26]. Given the regulatory role of Flt-1 in pregnancy and the presence of a multiallelic polymorphism in the Flt-1 gene, Flt-1 could conceivably be a candidate susceptibility gene in preeclampsia.

In this study, we focused on a d(TG)_n _repeat polymorphism in the Flt-1 gene which may be associated with preeclampsia in Korean pregnant women. First, the Flt-1 gene contains a polymorphic TG dinucleotide repeat in the 3' non-coding region that might affect signal transduction. Secondly, decreased Flt-1 expression has been shown in the placental bed of preeclamptic patients [27]. The down-regulation of Flt-1 in the placental bed may result in a decreased maternal vascular adaptation to pregnancy [27]. Third, the production of the soluble, alternatively spliced Flt-1, sFlt-1, is significantly higher in preeclamptic patients compared with normotensive pregnant women [28]. Finally, the embryos of Flt-1 mutant mice develop vasculature through endothelial cell differentiation. This vasculature is highly abnormal and unorganized with an overgrowth of endothelial cells crowding the lumen [29].

Polymeropoulos [14] first demonstrated that a higher percentage of 50 individuals from an American population had the 14-repeat allele of the (TG)_n _polymorphism in the 3' non-coding region of the Flt-1 gene. They also observed that the distribution of the (TG)_n _repeat alleles was bimodal, with two peaks at 14 repeats and 21 repeats, respectively. In this study, we tested, for the first time, the association between preeclampsia and the TG dinucleotide repeat polymorphism in the 3' non-coding region of the Flt-1 gene. Our data reveals that two alleles (14-repeat and 21-repeat) predominate in both preeclamptic and normal pregnancies, similar to the observation of Polymeropoulos [14]. However, we could not find any differences in the allele or genotype frequencies of Flt-1 between preeclamptic patients and normal controls. This polymorphism of the Flt-1 gene was also studied by Parry et al. in minimal change nephropathy (MCN) patients compared to the standard American population [19]. The investigators hypothesized that misregulation of Flt-1 may provide a mechanism for the development of protenuria in MCN, thus, polymorphisms in this gene may predispose to MNC. However, they did not demonstrate any deviation in the allele frequency in patients with MNC, implying that this locus does not contribute to susceptibility to MCN. Furthermore, we found that this dinucleotide polymorphism is not associated with a predisposition to preeclampsia. Alternatively, the penetrance of the Flt-1 gene may be modified by other factors, including distinct genetic loci that impart susceptibility.

Flt-1 can be activated by VEGF and placental growth factor (PlGF), which are highly expressed in the placenta. Most of the Flt-1 produced in the mouse and human placenta during later stages of gestation is the soluble form (sFlt-1) generated by alternative splicing of Flt-1, leading to a premature termination after the sixth Ig-like domain [30]. sFlt-1 binds both VEGF and PlGF and acts as a soluble antagonist of their action. Flt-1 misregulation in peripheral blood mononuclear cells of pregnant women can result in the over-expression of sFlt-1, which may produce an additional (extra-placental) source of sFlt-1 that contributes to the etiology of preeclampsia [31]. A recent report showed that levels of maternal sFlt-1 were elevated in preeclampsia and that administration of sFlt-1 to pregnant rats can cause symptoms of preeclampsia with glomerular endotheliosis [32]. This idea clearly suggests that placental Flt-1 can play roles in regulating maternal vasculature during pregnancy. We have previously shown that sFlt-1 levels in the second trimester maternal plasma are significantly higher in women with preeclampsia than in normal pregnant women [33]; however, we were unable to demonstrate an association between the d(TG)_n _polymorphism in the 3' non-coding region of the Flt-1 gene and sFlt-1 levels. 

Most common genetic disorders, such as preeclampsia, follow a complex mode of inheritance and may result from variants of many genes, each contributing only a weak effect to the disease. Common genetic polymorphism may explain a portion of the heritable risk for common diseases. Consequently considerable effort should be devoted to finding and typing common microsatellite polymorphisms in the human genome in order to understand the occurrence of relatively common phenotypes. As trophoblast cells, which also express Flt-1, are fetal of origin, the role of fetal Flt-1 (TG)_n _polymorphism needs also to be examined in the risk of preeclampsia. Although we do not deny that one of the possible limitations of this case-control study is the relatively small sample size, we did take into account several issues that could lead to a false conclusion, such as established criteria for the diagnosis in order to exclude subphenotypes known to differ in the evaluation of the disease [34] and matching of cases and controls for several risk factors and for genetic background.

## Conclusion

To our knowledge, this is the first study investigating the association between the d(TG)_n _repeat polymorphism in the 3' non-coding region of the human Flt-1 gene [14,15,19] and preeclampsia. In the present study, allele and genotype frequencies of this dinucleotide repeat polymorphism were not different between preeclamptic and normal pregnancies. Our results suggest that the Flt-1 (TG)_n _polymorphism is not associated with susceptibility to the development of preeclampsia in Korean pregnant women. This result might be indicative of the large diversity in the genetic background of preeclampsia, although this observation deserves further analysis in a larger group of preeclamptic patients.

## Competing interests

The authors declare that they have no competing interests.

## Authors' contributions

S–YK conceived the study, performed sequencing of the samples, and contributed to the analysis and interpretation of the data as well as to the writing of the manuscript. J–HL collected relevant clinical data and assisted in preparation of the DNA samples. J–HY, M–YK and JSC participated in collection of samples, clinical analyses, and clinical diagnosis. S–YP participated in collection of samples and revised the manuscript. H–MR developed the study design, was responsible for overall supervision of all aspects of this research project and revised the manuscript. All authors read and approved the final manuscript.

## Pre-publication history

The pre-publication history for this paper can be accessed here:



## References

[B1] Brosens IA (1977). Morphological changes in the utero-placental bed in pregnancy hypertension. Clin Obstet Gynaecol.

[B2] Roberts JM, Taylor RN, Musci TJ, Rodgers GM, Hubel CA, McLaughlin MK (1989). Preeclampsia: an endothelial cell disorder. Am J Obstet Gynecol.

[B3] Redman CW, Sargent IL (2000). Placental debris, oxidative stress and pre-eclampsia. Placenta.

[B4] Meis PJ, Goldenberg RL, Mercer BM, Iams JD, Moawad AH, Miodovnik M, Menard MK, Caritis SN, Thurnau GR, Bottoms SF, Das A, Roberts JM, McNellis D (1998). The preterm prediction study: risk actors for indicated preterm births. Maternal-Fetal Medicine Units Network of the National Institute of hild Health and Human Development. Am J Obstet Gynecol.

[B5] Ahmed A, Perkins J (2000). Angiogenesis and intrauterine growth restriction. Baillieres Best Pract Res Clin Obstet Gynaecol.

[B6] Ferrara N (1999). Molecular and biological properties of vascular endothelial growth factor. J Mol Med.

[B7] Yancopoulos GD, Davis S, Gale NW, Rudge JS, Wiegand SJ, Holash J (2000). Vascular-specific growth factors and blood vessel formation. Nature.

[B8] Hiratsuka S, Minowa O, Kuno J, Noda T, Shibuya M (1998). Flt-1 lacking the tyrosine kinase domain is sufficient for normal development and angiogenesis in mice. Proc Natl Acad Sci USA.

[B9] Fong GH, Rossant J, Gertsenstein M, Breitman ML (1995). Role of the Flt-1 receptor tyrosine kinase in regulating the assembly of vascular endothelium. Nature.

[B10] Clark DE, Smith SK, Sharkey AM, Charnock-Jones DS (1996). Localization of VEGF and expression of its receptors flt and KDR in human placenta throughout pregnancy. Hum Reprod.

[B11] Shibuya M, Yamaguchi S, Yamane A, Ikeda T, Tojo A, Matsushime H, Sato M (1990). Nucleotide sequence and expression of a novel human receptor-type tyrosine kinase gene (flt) closely related to the fms family. Oncogene.

[B12] Kondo K, Hiratsuka S, Subbalakshmi E, Matsushime H, Shibuya M (1998). Genomic organization of the flt-1 gene encoding for vascular endothelial growth factor (VEGF) receptor-1 suggests an intimate evolutionary relationship between the 7-Ig and the 5-Ig tyrosine kinase receptors. Gene.

[B13] Kendall RL, Thomas KA (1993). Inhibition of vascular endothelial cell growth factor activity by an endogenously encoded soluble receptor. Proc Natl Acad Sci USA.

[B14] Polymeropoulos MH, Rath DS, Xiao H, Merril CR (1991). Dinucleotide repeat polymorphism at the human fms-related tyrosine kinase gene (FLT1). Nucleic Acids Res.

[B15] Han HJ, Fujiwara T, Shin S, Nakamura Y (1993). Dinucleotide repeat polymorphism in the 3' non-coding region of the FLTI gene. Hum Mol Genet.

[B16] Freimer NB, Slatkin M (1996). Microsatellites: evolution and mutational processes. Ciba Found Symp.

[B17] Wang E, Ma WJ, Aghajanian C, Spriggs DR (1997). Posttranscriptional regulation of protein expression in human epithelial carcinoma cells by adenine-uridine-rich elements in the 3'-untranslated region of tumor necrosis factor-alpha messenger RNA. Cancer Res.

[B18] Cuningham FG, Gant NF, Leveno KJ, Gilstrap LC, Hauth JC, Wenstrom KD (2001). Williams Obstetrics.

[B19] Parry RG, Gillespie KM, Clark AG, Mathieson PW (1999). Dinucleotide repeat polymorphisms within the Flt-1 gene in minimal change nephropathy. Eur J Immunogenet.

[B20] Gabellini N (2001). A polymorphic GT repeat from the human cardiac Na+Ca2+ exchanger intron 2 activates splicing. Eur J Biochem.

[B21] Gebhardt F, Zänker KS, Brandt B (1999). Modulation of epidermal growth factor receptor gene transcription by a polymorphic dinucleotide repeat in intron 1. J Biol Chem.

[B22] Turecki G, Grof P, Cavazzoni P, Duffy A, Grof E, Ahrens B, Berghöfer A, Müller-Oerlinghausen B, Dvoráková M, Libigerová E, Vojtechovský M, Zvolský P, Joober R, Nilsson A, Prochazka H, Licht RW, Rasmussen NA, Schou M, Vestergaard P, Holzinger A, Schumann C, Thau K, Rouleau GA, Alda M (1998). Evidence for a role of phospholipase C-gamma1 in the pathogenesis of bipolar disorder. Mol Psychiatry.

[B23] Epplen C, Santos EJ, Guerreiro JF, van Helden P, Epplen JT (1997). Coding versus intron variability: extremely polymorphic HLA-DRB1 exons are flanked by specific composite microsatellites, even in distant populations. Hum Genet.

[B24] Roberts JM, Cooper DW (2001). Pathogenesis and genetics of pre-eclampsia. Lancet.

[B25] Jakeman LB, Winer J, Bennett GL, Altar CA, Ferrara N (1992). Binding sites for vascular endothelial growth factor are localized on endothelial cells in adult rat tissues. J Clin Invest.

[B26] Peters KG, De Vries C, Williams LT (1993). Vascular endothelial growth factor receptor expression during embryogenesis and tissue repair suggests a role in endothelial differentiation and blood vessel growth. Proc Natl Acad Sci USA.

[B27] Tsatsaris V, Goffin F, Munaut C, Brichant JF, Pignon MR, Noel A, Schaaps JP, Cabrol D, Frankenne F, Foidart JM (2003). Overexpression of the soluble vascular endothelial growth factor receptor in preeclamptic patients: pathophysiological consequences. J Clin Endocrinol Metab.

[B28] Koga K, Osuga Y, Yoshino O, Hirota Y, Ruimeng X, Hirata T, Takeda S, Yano T, Tsutsumi O, Taketani Y (2003). Elevated serum soluble vascular endothelial growth factor receptor 1 (sVEGFR-1) levels in women with preeclampsia. J Clin Endocrinol Metab.

[B29] Hiratsuka S, Maru Y, Okada A, Seiki M, Noda T, Shibuya M (2001). Involvement of Flt-1 tyrosine kinase (vascular endothelial growth factor receptor-1) in pathological angiogenesis. Cancer Res.

[B30] He Y, Smith SK, Day KA, Clark DE, Licence DR, Charnock-Jones DS (1999). Alternative splicing of vascular endothelial growth factor (VEGF)-R1 (FLT-1) pre-mRNA is important for the regulation of VEGF activity. Mol Endocrinol.

[B31] Rajakumar A, Michael HM, Rajakumar PA, Shibata E, Hubel CA, Karumanchi SA, Thadhani R, Wolf M, Harger G, Markovic N (2005). Extra-placental expression of vascular endothelial growth factor receptor-1, (Flt-1) and soluble Flt-1 (sFlt-1), by peripheral blood mononuclear cells (PBMCs) in normotensive and preeclamptic pregnant women. Placenta.

[B32] Maynard SE, Min JY, Merchan J, Lim KH, Li J, Mondal S, Libermann TA, Morgan JP, Sellke FW, Stillman IE, Epstein FH, Sukhatme VP, Karumanchi SA (2003). Excess placental soluble fms-like tyrosine kinase 1 (sFlt1) may contribute to endothelial dysfunction, hypertension, and proteinuria in preeclampsia. J Clin Invest.

[B33] Kim SY, Ryu HM, Yang JH, Kim MY, Han JY, Kim JO, Chung JH, Park SY, Lee MH, Kim DJ (2007). Increased sFlt-1 to PlGF ratio in women who subsequently develop preeclampsia. J Korean Med Sci.

[B34] (2000). Report of the National High Blood Pressure Education Program Working Group on High Blood Pressure in Pregnancy. Am J Obstet Gynecol.

